# Is netrin-1 a reliable inflammatory marker for periodontitis?

**DOI:** 10.34172/japid.2022.001

**Published:** 2022-01-24

**Authors:** Sarah Yasser Abdulfattah, Azza Abdel Baky Baiomy, Jilan Mohammed Youssef

**Affiliations:** ^1^Department of Oral Medicine, Periodontology, Diagnosis and Oral Radiology, Faculty of Dentistry, Mansoura University, Mansoura, Egypt; ^2^Department of Clinical Pathology, Faculty of Medicine, Mansoura University, Mansoura, Egypt

**Keywords:** Gingival crevicular fluid, host response, inflammation, netrin-1, periodontitis, scaling and root planing

## Abstract

**Background:**

The current study tested netrin-1 as a reliable inflammatory marker of periodontal disease.

**Methods:**

Gingival crevicular fluid (GCF) samples were taken at baseline from 30 systemically healthy individuals. Fifteen subjects had stage II grade A or B periodontitis, and 15 were periodontally and clinically healthy. Whole-mouth periodontal parameters [probing depth (PD), clinical attachment loss (CAL), plaque index (PI), and gingival index (GI)] were recorded. The GCF samples were re-collected, and clinical parameters were re-recorded after six weeks following full-mouth scaling and root planing (SRP) in the periodontitis group.

**Results:**

Netrin-1 GCF levels were significantly lower in periodontitis patients than periodontally healthy individuals at baseline with a significant increase in netrin-1 GCF levels after SRP (*P*<0.05).

**Conclusion:**

Netrin-1 may have a significant role in the inflammatory process of chronic periodontitis; thus, it could be a promising anti-inflammatory marker in periodontal disease.

## Introduction

 Chronic periodontitis represents a biological process of the host response dysregulation and microbial dysbiosis in periodontal tissues.^1^ The role of pathogenic bacteria in periodontal disease has been limited to the keystone hypothesis, stating that specific pathogens modify the host response to disrupt immunity and change the balance from bacterial homeostasis to bacterial dysbiosis.^2^ Although the dental plaque microorganisms are blamed for the initiation of periodontal inflammation, the host immune response against these microorganisms, including the production and release of inflammatory mediators, are mainly, if not completely, responsible for periodontal breakdown.^3^

 The host immune-inflammatory reaction in periodontitis is considered the basic determinant of disease pattern, severity, and progression. As the disease progresses, the immune system responds by producing inflammatory cytokines, including interleukins (IL), tumor necrosis factor-a (TNF-a), prostanoids such as prostaglandin E2 (PGE2), and enzymes such as the matrix metalloproteinases (MMPs). The levels of inflammatory mediators in the periodontal tissues are generally balanced through the enzymes and cytokines of the host immune system, which finally function together to eliminate microbial insult and protect the host.^4,5^

 Improper immune response or inability of the immune system to control the inflammatory reaction results in the overproduction of destructive enzymes and inflammatory cytokines. However, many acquired and environmental risk factors could also aggravate the host’s inflammatory response and create an imbalance between the pro-inflammatory and anti-inflammatory mediators inside the periodontal tissues, finally leading to tissue destruction. Generally, this shift in paradigms considering periodontitis as inflammatory, not an infectious disease, has resulted in the development of host modulatory therapies in the field of perioceutics.^6,7^

 Netrin-1 is a laminin-related protein originally described as a diffusible molecule secreted from the ventral structure in the developing spinal cord and recognized as a neuro-immune guidance cue.^8^ It is a classical neuronal leading cue that was widely investigated to maintain inflammatory microenvironment hemostasis. It was also found to be relevant to proangiogenic processes.^9^

 Five netrins have been identified in vertebrates.^10^ Netrin-1 is the most studied among all the netrins, which is also expressed outside the nervous system. Relative quantification of mRNA indicates elevated levels of netrin-1 in the heart, brain, lungs, and kidneys. It is also related to inﬂammation and tumorigenesis in adulthood.^11,12^

 Netrin-1 functions in both acute and chronic inflammatory conditions by regulating angiogenesis, immune cell recruitment, and macrophage reprogramming, in addition to regulating PGE2 production mediated by cyclooxygenase-2 (COX-2). There is evidence that netrin-1 modifies inflammation through its impact on immune cells by regulating their recruitment, motility, stimulation, and cytokine production.^13^

 Several studies have reported that macrophages and monocytes are the main components of the host response, as their retention in the periodontal tissues leads to prolonged chronic inflammation.^14^ Several studies have demonstrated that netrin-1 can modify the migration of these cells and restricts the transmigration into the tissues in inflammation through its receptor Unc5b.^15^

 Furthermore, netrin-1 modulates the inflammatory response of macrophages by inhibiting COX-2–mediated PGE2 production and inducing macrophage reprogramming to promote the resolution of inflammation.^16^

 Moreover, netrin-1 participates in osteoclast biology by binding to its receptor UNC5b, and therefore, has been suggested as a therapeutic target for inflammatory bone destructive diseases.^17^

 This study was designed to assess gingival crevicular fluid (GCF) netrin-1 levels in chronic periodontitis to test its validity as an inflammatory marker in periodontal disease.

## Methods

###  Subjects 

 This study was conducted on 30 systemically healthy individuals, 15 of which are periodontally healthy (healthy group; 37.13±7.37 years of age), and the other 15 suffered from stage II grade A or B periodontal disease (study group; 43.13±9.26 years of age). The participants were selected from the outpatient clinic of the Department of Periodontology, Faculty of Dentistry, Mansoura University. All the procedures, steps, aims, possible outcomes, and potential hazards of the study were well understood and accepted by all the participants. All the participants agreed to sign an informed consent form to be included in the study. The Ethics Committee of Mansoura Faculty of Dentistry approved the study protocol under the code: A 1 0 1 4 0 4 2 0.

###  Inclusion criteria

 Patients >20 years of age with stage II grade A or B periodontitis with clinical attachment loss (CAL) of 3-4 mm, and a probing depth of >4 mm. They also had no history of antibiotic administration or had received no periodontal therapy in the last three months.

###  Exclusion criteria

 Patients with systemic conditions that could influence the progression of periodontitis or impair the treatment outcome (e.g., diabetes mellitus) were excluded; in addition, smokers and pregnant and lactating women were excluded. Also, individuals undergoing current and long-term administration of anti-inflammatory medications were excluded.

###  Study design

 A case-control study was conducted. The control group consisted of 15 periodontally healthy participants, and the study group consisted of 15 periodontitis patients.

###  Treatment phase

 At baseline, probing depth (PD),^18^ measured from the free gingival margin to the depth of the pocket, and clinical attachment loss (CAL),^19^ measured from the cementoenamel junction to the base of the pocket, were recorded at six aspects of the tooth of interest in each patient (mesiofacial, mid-facial, distofacial, mesiolingual, mid-lingual, and distolingual), using a UNC-15 probe inserted parallel to the long axis of the tooth. The plaque index (PI)^20^ and gingival index (GI)^21^ were evaluated at four aspects of the teeth of interest: distofacial dental papilla, mesiofacial dental papilla, facial gingival margins, and the whole lingual gingival margin. Subsequently, GCF samples were collected from the deepest pocket with sterile paper points inserted for 30 seconds. GCF samples were then placed in Eppendorf tubes and stored at −80°C until the time of analysis. Periodontitis patients were subjected to full-mouth scaling and root planing (SRP) by a periodontist (YS), using ultrasonic scalers the day after sample collection. SRP was repeated weekly if indicated, and patients were instructed to maintain good oral hygiene measures. Six weeks after the last session of SRP, periodontal parameters were reevaluated, and GCF samples were recollected from periodontitis patients. Periodontal examinations were performed by two qualified periodontists (YS and YMJ), with an interval of 1 to 2 hours between the two examinations of the same individual, and measurements were within the range of 1 mm of change with 95% confidence level.

###  Laboratory analysis

 Netrin-1 was assayed by sandwich ELISA supplied by Bioassay Technology Laboratory (China) according to the manufacturer’s instructions.

**Figure 1:**
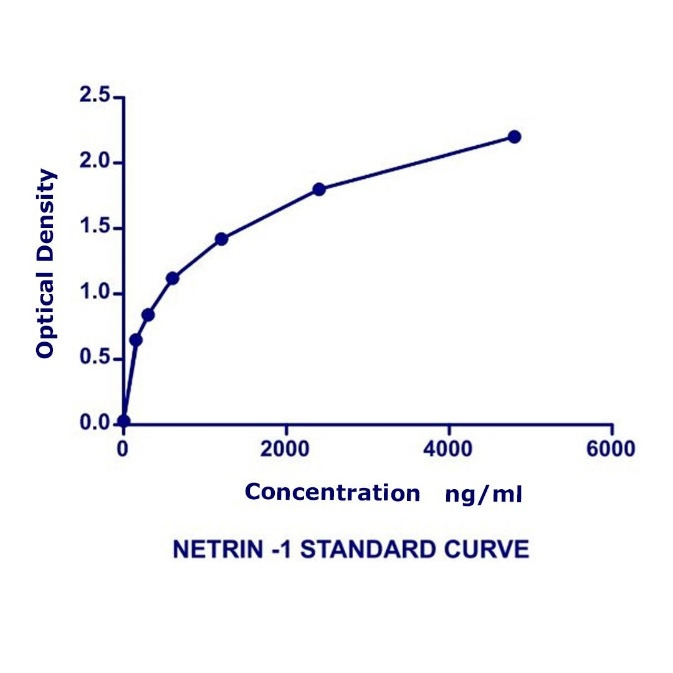
Netrin-1 standard curve relating the optical density to the concentration of the standards.

 Netrin-1 ELISA kit applies the quantitative sandwich enzyme immunoassay technique. The microtiter plate has been pre-coated with a specific monoclonal antibody. Standards and samples were then added to the microtiter plate wells, and netrin-1, if present, would bind to the antibody pre-coated wells. To quantitatively determine the amount of netrin-1 present in the sample, a standardized preparation of horseradish peroxidase-conjugated polyclonal antibody, specific for netrin-1, was added to each well to “sandwich” the netrin-1 immobilized on the plate. The microtiter plate incubated at 37°C for 60 minutes, and then the wells were thoroughly washed with distilled water to remove all unbound components. Next, substrate solutions were added to each well. The enzyme HRP and substrate were allowed to react over a short incubation period at 37ºC. Only those wells that contained netrin-1 and enzyme-conjugated antibodies would exhibit a color change. The enzyme-substrate reaction was terminated by adding a sulphuric acid solution, and the optical density (OD) was measured spectrophotometrically at a wavelength of 450 nm. A standard curve was plotted relating the OD to the concentration of standards. The netrin-1 concentration in each sample was interpolated from this standard curve (Figure 1). GCF netrin-1 level was estimated per 30 seconds as the paper point was inserted for 30 seconds.

###  Statistical analysis

 The data were computed and treated for statistical analysis at 5% level of significance, using SPSS 20.0. In the description of quantitative data, means and standard deviations were used for parametric data. Shapiro-Wilk test was used for normality assessment of both groups.

## Results

###  Demographic data

Table 1 summarizes the demographic characteristics of the two groups. There was no statistically significant difference between the study and control groups regarding age and gender, with mean ages of 43.13 and 37.13 in the study and control groups, respectively. Male gender was more frequent in both the study and control groups by 53.3% and 73.3%, respectively.

**Table 1:** Sociodemographic characteristics of the studied groups

**Parameter**	**Study group** **N=15**	**Control group** **N=15**	**Test of significance**
**Age/years**	43.13±9.26	37.13±7.37	t=1.96 p=0.06
**Sex** **Male** **Female**	8(53.3%) 7(46.7%)	11(73.3%) 4(26.7%)	χ^2^=1.29 p=0.256

t: Student’s t-test χ2: Chi-squared test

 Clinical and laboratory findings

 Regarding intragroup comparison in the study group, there were significant improvements in all the clinical parameters of GI, PI, PD, and CAL when the mean values were compared six weeks after treatment with the baseline values (P<0.001). However, regarding intergroup comparison, there were significant differences between study and control groups regarding PI, GI, PD, and CAL, at both baselines and after-treatment values (Table 2).

**Table 2:** Comparison of clinical parameters before and after treatment among studied groups

**Parameter**		**Study group** **N=15**	**Control group** **N=15**	**Student t test**
**PI**	Before	2.54±0.216	0.0±0.0	t=45.51 P<0.001*
	After	0.788±0.125	0.0±0.0	t=24.32 P<0.001*
**Paired t-test**		t=37.48 P<0.0001*	t=0 P=1.0	
**GI**	Before	1.80±0.14	0.0±0.0	t=49.70 P<0.001*
	After	0.369±0.067	0.0±0.0	t=21.34 P<0.001*
**Paired t-test**		t=37.48 P<0.001*	t=0 P=1.0	
**PD**	Before	3.53±0.255	1.40±0.183	t=26.21 P<0.001*
	After	1.69±0.19	1.40±0.183	t=4.19 P<0.001*
**Paired t-test**		t=57.95 P<0.001*	t=0 P=1.0	
**CAL**	Before	2.63±0.317	0.0±0.0	t=32.11 P<0.001*
	After	1.47±0.24	0.0±0.0	t=23.73 P<0.001*
**Paired t-test**		t=23.79 P<0.001*	t=0 P=1.0	
**Netrin-1**	Before	12.29±0.62	28.56±0.99	t=53.43 P<0.001*
	After	23.38±0.78	28.56±0.99	t=15.75 P<0.001*
**Paired t-test**		t=43.23 P<0.001*	t=0 P=1.0	

t: Student’s t-test *Statistically significant (P<0.05). Abbreviations: PI, plaque index; GI, gingival index; PD, probing depth; CAL, clinical attachment loss.

 Regarding baseline values of GCF netrin-1 levels, there were statistically significant differences between the study and control groups, with markedly lower values in the study group. Six weeks after treatment, there was a statistically significant increase in mean GCF netrin-1 levels in the study group compared to baseline values (P<0.001). However, no significant correlation was noticed between GCF netrin-1 level and the clinical indices tested before and after treatment (Table 3).

**Table 3:** Correlation between netrin-1 and clinical indices

**Parameter**	**Netrin-1**	
	**Before**	**After**
**PI**	r=0.265 P=0.340	r=0.271 P=0.328
**GI**	r=-0.179 P=0.523	r=0.146 P=0.605
**PD**	r=-0.191 P=0.496	r=0.488 P=0.065
**CAL**	r=0.112 P=0.690	r=0.042 P=0.881

r: Pearson’s correlation coefficient Abbreviations: PI, plaque index; GI, gingival index; PD, probing depth; CAL, clinical attachment loss.

## Discussion

 The present study was undertaken to investigate the impact of periodontitis and SRP on GCF netrin-1 levels, testing it as a novel and reliable periodontal inflammatory marker. To the best of our knowledge, we are among the first to test the role of netrin-1 in periodontal disease and health.

 GCF netrin-1 levels were markedly lower in periodontitis patients than in healthy ones in the present study. Six weeks after SRP, there was a significant increase in GCF netrin-1 level in periodontitis patients, although it was significantly lower than the control value. This finding may be due to the relatively short follow-up period.

 In contrast to our results, Gunpinar et al^22^ reported higher GCF netrin-1 levels in chronic periodontitis patients than in healthy individuals. Furthermore, those levels decreased significantly after periodontal therapy. They explained it because netrin-1 inhibits macrophage migration, leading to the retention of tissue macrophages and the promotion of chronic inflammation.

 In this regard, an in vitro and in vivo study by Ly et al^11^ revealed that infection and pro-inflammatory models significantly decreased the level of macrophage-derived netrin-1, promoting the attraction of leukocytes to the site of inflammation. They hypothesized that netrin-1 is an immune-modulator that maintains leukocyte levels in check, preventing aggressive tissue destruction.

 Another in vivo study on primary human aortic endothelial cells and human monocytes by Lin et al^23^ showed that netrin-1 and its receptor Unc5b had an anti-inflammatory effect on endothelial cells, which was markedly inhibited by the exogenous administration of TNF-α.

 Additionally, Passacquale et al^24^ reported that netrin-1 is downregulated by infection and pro-inflammatory cytokines, leading to a decrease in netrin-1 protective effects on endothelial cells, which accelerates inflammation progression. Also, Bruikman et al^25^ demonstrated that the plasma levels of netrin-1 are inversely related to arterial wall inflammation in atherosclerotic plaques.

 Other researchers described netrin-1 as a pro-inflammatory mediator. Van Gils et al^26^ reported that netrin-1 might enhance atherosclerosis via suppressing foam calls’ migration to the lymph nodes, being trapped in the atherosclerotic plaques, and participating in its development and instability. They also reported that lack of netrin-1 in the macrophages decreased atherosclerosis and promoted the migration of foam cells to the lymphatic system.

 Likewise, Ramkhelawon et al^9^ demonstrated in accordance with what was reported in the mutant mice model that netrin-1 enhances the accumulation of macrophages in adipose tissue, increasing metabolic disturbance and inflammatory reactions.

 Consistent with our results, Ranganathan et al^27^ and Mao et al^16^ disagreed with what was reported by Ramkhelawon et al^9^ regarding macrophage phenotypes. The results indicated that a high level of netrin-1 was protective, promoting macrophages to shift to M2 phenotype, in conjunction with increased anti-inflammatory cytokines levels, such as IL-13 and IL-4, and decreased COX-2 and IL-6 levels. They also reported that netrin-1 stimulates anti-inflammatory pathways responsible for the regulation of macrophage polarization, like peroxisome proliferator-activated receptors (PPAR), which modulates many inflammatory mechanisms, including inhibiting the activity of necrosis factor-kappa B (NF-κB). They also showed that netrin-1 decreased the polarization of M1 and the production of cytokines induced by suppressed ischemia-reperfusion injury and interferon-gamma (IFN-γ), while these effects were inhibited by PPAR antagonists.

 Another in vitro and in vivo study by Ranganathan et al^13^ suggested that netrin-1 modulates inflammatory reactions by suppressing NF-κB, causing downregulation of COX-2-mediated production of PGE2 and thromboxane A2.

 As our study is a preliminary study, we recommend further studies with a larger population and a longer follow-up period to evaluate the relation between netrin-1 and other inflammatory cytokines and test the role of netrin-1 and its receptor UNC5b in the inflammatory process.

## Conclusions

 Estimation of netrin-1 GCF level showed significantly lower levels in periodontitis patients than periodontally healthy individuals. After periodontal treatment, netrin-1 GCF levels increased significantly compared to baseline measurements. Based on these findings, we suggest that netrin-1 could be considered a potential anti-inflammatory biomarker of periodontal disease.

## Authors’ contributions

 YS: This author planned the study design and was responsible for the clinical work of the study, including patient selection, treatment, and follow-up, and collection of data. BAA: This author was responsible for the laboratory work and data analysis and interpretation. YMJ: This author was responsible for drafting the manuscript and publishing it. BAA and YMJ critically received the manuscript. All the authors approved the final submission.

## Funding

 There was no financial support for the study.

## Availability of data

 The data that support our results are available if requested from the corresponding author, YMJ. However, the data are not available for the public, as they contain private information about research volunteers

## Ethics approval

 Ethical approval number: A10140420

## Competing interests

 The authors declare that they have no competing interest.
